# Supplementary material for: Lead Time and Prognostic Role of Serum CEA, CA19-9, IL-6, CRP, and YKL-40 after Adjuvant Chemotherapy in Colorectal Cancer
Source: Cancers (Basel). 2021 Aug 2;13(15):3892. doi: 10.3390/cancers13153892 (PMC8345682; doi:10.3390/cancers13153892)
Supplement: Supplementary file 1 [file cancers-13-03892-s001.zip › cancers-1298988-supplementary.pdf]

A

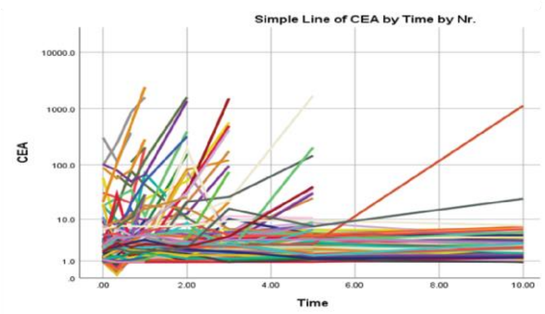

Biomarker	Time-point	Median	Percentile 25	Percentile 75
CEA	0 months (postoperative)	1.9	1.2	3.0
	4 months (during adjuvant)	2.6	1.8	4.2
	8 months (post-adjuvant)	2.5	1.8	3.8
	12 months	2.8	1.9	4.7
	24 months	2.7	2.0	4.8
	36 months	2.7	1.9	4.8
	60 months	2.7	1.6	4.7
	120 months	2.3	1.5	3.5

B

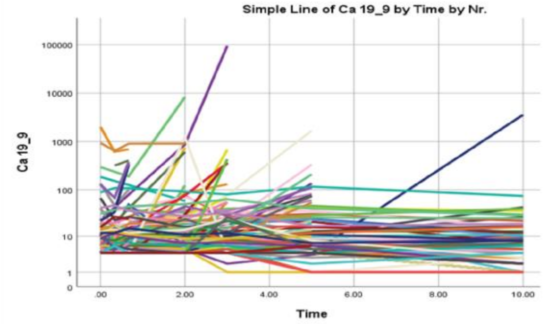

Biomarker	Time-point	Median	Percentile 25	Percentile 75
CA19-9	0 months (postoperative)	4	4	9
	4 months (during adjuvant)	6	4	14
	8 months (post-adjuvant)	6	4	14
	12 months	6	4	14
	24 months	7	4	16
	36 months	8	4	22
	60 months	7	4	20
	120 months	6	3	12

C

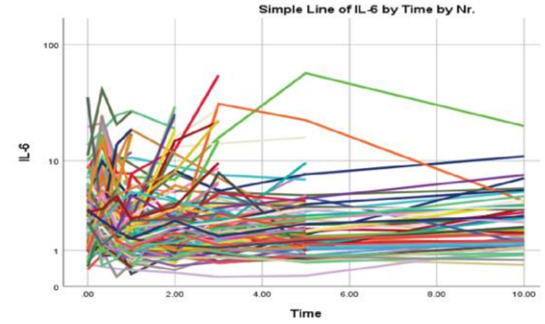

Biomarker	Time-point	Median	Percentile 25	Percentile 75
IL-6	0 months (postoperative)	2.3	1.6	3.5
	4 months (during adjuvant)	2.7	1.7	4.2
	8 months (post-adjuvant)	1.9	1.4	3.3
	12 months	2.1	1.3	3.1
	24 months	2.1	1.3	3.5
	36 months	2.1	1.4	3.5
	60 months	2.0	1.2	3.4
	120 months	2.3	1.4	3.7

D

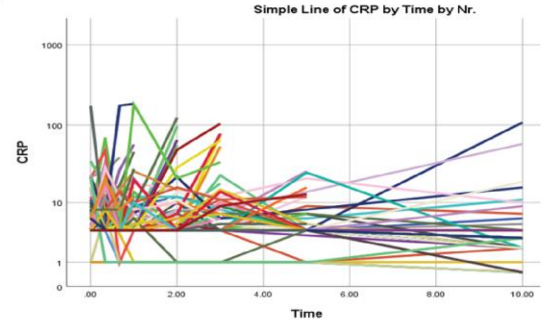

Biomarker	Time-point	Median	Percentile 25	Percentile 75
CRP	0 months (postoperative)	4	4	7
	4 months (during adjuvant)	4	4	7
	8 months (post-adjuvant)	4	4	5
	12 months	4	4	5
	24 months	4	4	6
	36 months	4	4	7
	60 months	4	4	4
	120 months	3	2	3

E

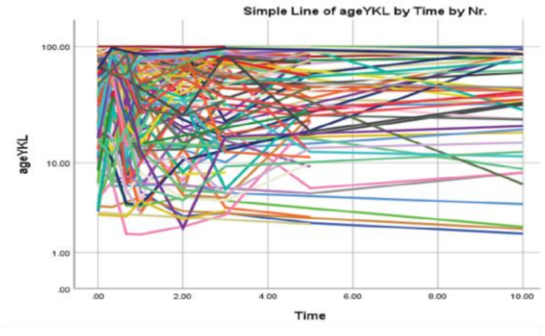

Biomarker	Time-point	Median	Percentile 25	Percentile 75
YKL-40	0 months (postoperative)	65	39	119
	4 months (during adjuvant)	91	57	152
	8 months (post-adjuvant)	71	38	128
	12 months	60	37	108
	24 months	62	34	125
	36 months	63	31	116
	60 months	58	39	105
	120 months	63	38	124

Figure S1. Biomarker levels during 10 years of follow-up in each patient: CEA (A), CA 19-9 (B), IL-6 (C), CRP (D), and YKL-40 (E).

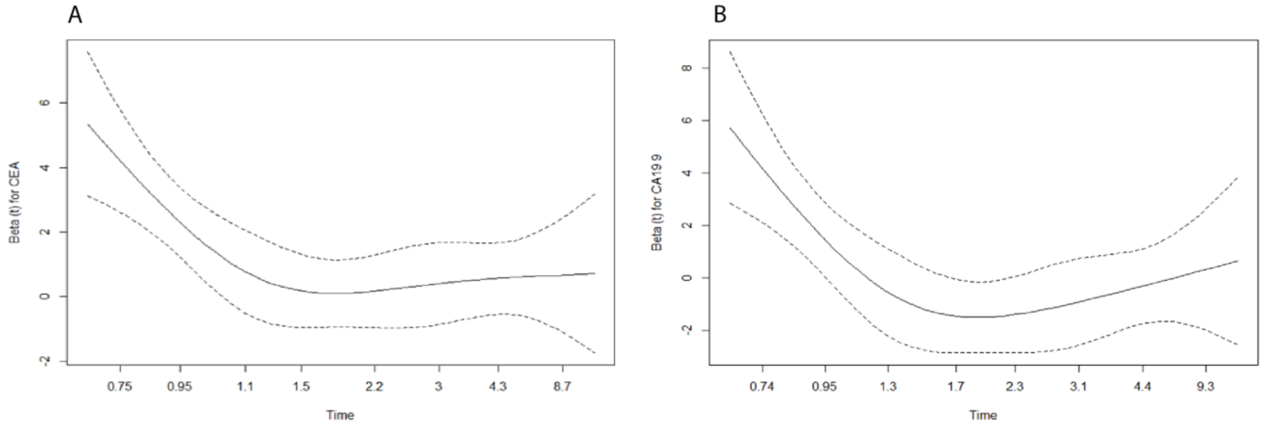

Figure S2. Schoenfeld residuals for CEA (A) and CA 19-9 (B) in a modified model for DFS.

Table S1. Modified model for DFS of CEA and CA19-9 with the time axis split in two at 12 months: for the first year after primary surgery and for the time after that.

	DFS		
	HR	95% CI for HR	P-value
**Adjusted***			
CEA[1] elevated vs. normal for 0-1 year	18.50	6.34—53.95	<0.001
CEA[1] elevated vs. normal for >1 year	1.19	0.26—5.39	0.824
CA19-9[2] elevated vs. normal for 0-1 year	6.75	2.15—21.15	0.001
CA19-9[2] elevated vs. normal >1 year	0.26	0.04—1.94	0.190

* Adjusted with age, sex, inflammatory disease, chemo regimen and TNM stage
[1] n= 130, [2] n= 119

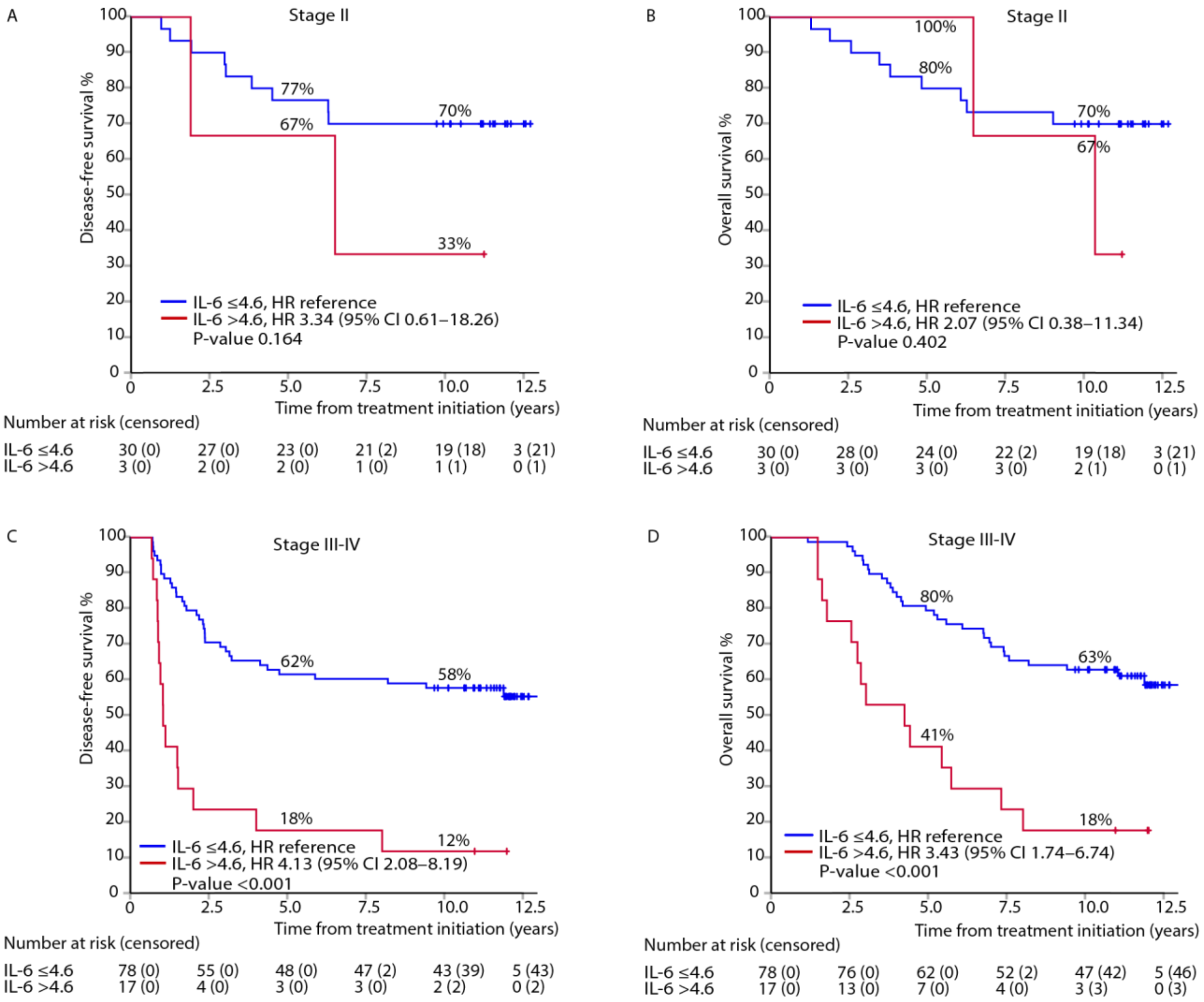

Figure S3. Disease-free survival (DFS) and overall survival (OS) after therapy in patients with elevated versus normal IL-6 and TNM stage II (A, B) or III–IV (C. D), adjusted hazard ratios (HR) and 95% confidence intervals (95% CI).

Table S2. Mutually adjusted, modified model for DFS of CEA and CA19-9 with the time axis was split in two at 12 months: for the first year after primary surgery and for the time after that.

	DFS		
	HR	95% CI for HR	P-value
**Adjusted with TNM stage, n=112**			
CEA elevated vs. normal for 0-1 year	8.61	2.32—31.94	0.001
CEA elevated vs. normal for >1 year	0.53	0.07—3.97	0.533
CA19-9 elevated vs. normal for 0-1 year	6.11	1.65—22.64	0.007
CA19-9 elevated vs. normal >1 year	0.25	0.03—1.84	0.172
IL-6 elevated vs. normal	2.39	0.98—5.81	0.056
CRP elevated vs. normal	1.41	0.50—3.99	0.521
YKL-40 elevated vs. normal	1.10	0.60—2.00	0.761

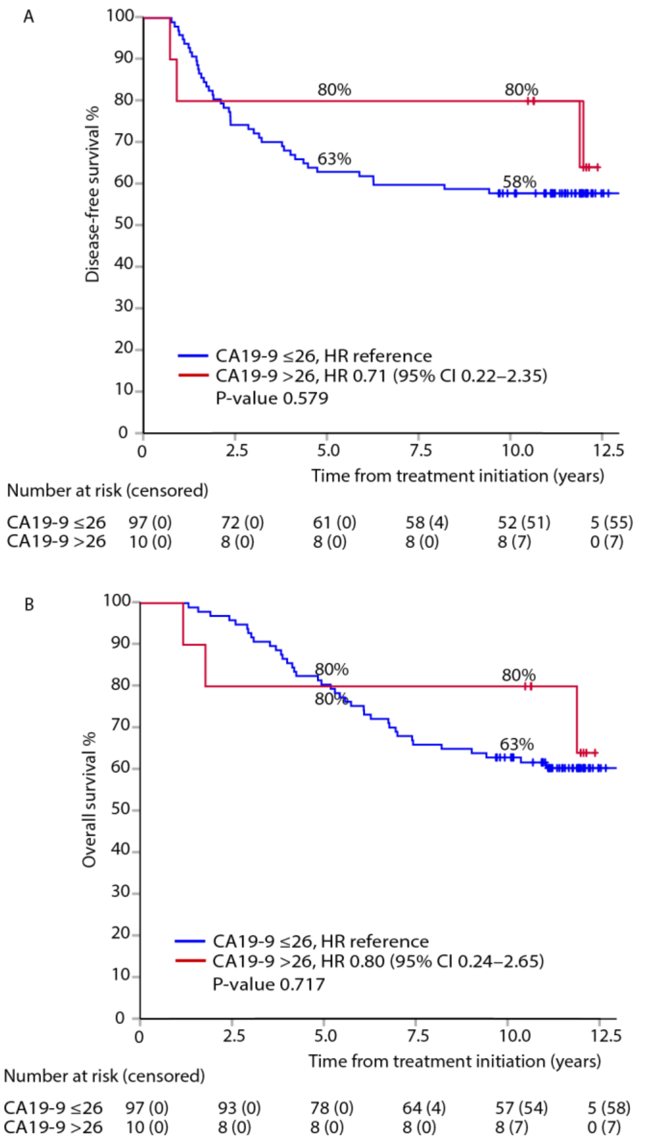

Figure S4. Association between elevated CA19-9 and outcomes: DFS (A) and OS (B), in the subgroup of patients with normal post-adjuvant CEA, adjusted hazard ratios (HR) and 95% confidence intervals (95% CI).

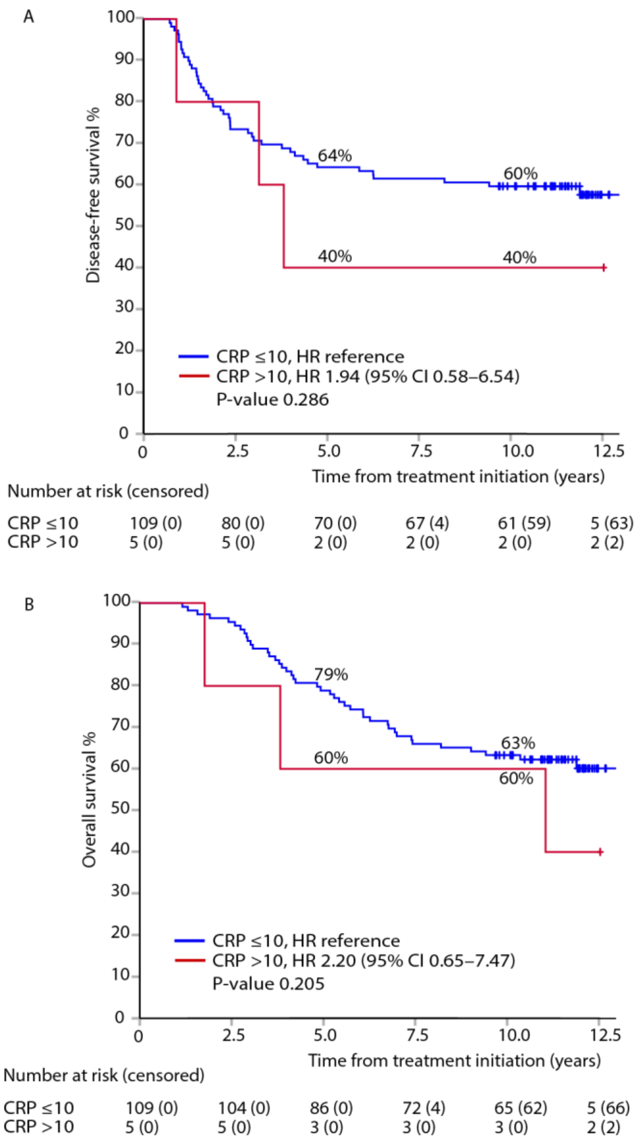

Figure S5. Association between elevated CRP and outcomes: DFS (A) and OS (B), in the subgroup of patients with normal post-adjuvant CEA, adjusted hazard ratios (HR) and 95% confidence intervals (95% CI).

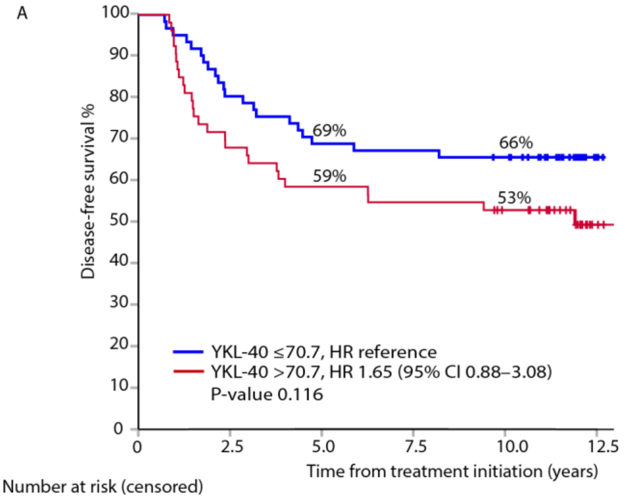

Number at risk (censored)

	0	2.5	5.0	7.5	10.0	12.5
YKL-40 ≤70.7	61 (0)	49 (0)	42 (0)	41 (1)	39 (37)	3 (40)
YKL-40 >70.7	53 (0)	36 (0)	31 (0)	29 (3)	25 (23)	4 (26)

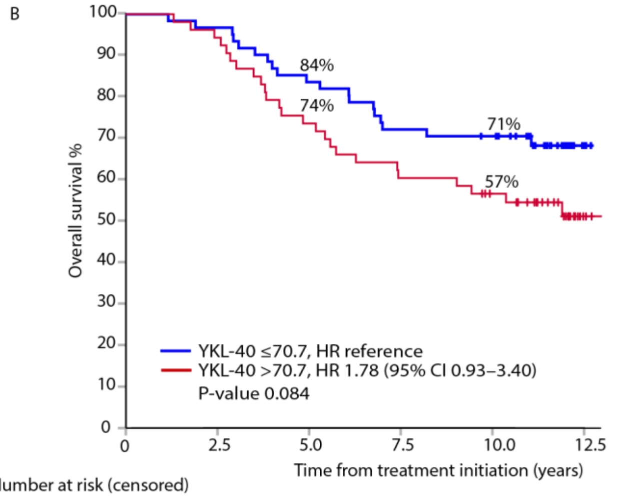

Number at risk (censored)

	0	2.5	5.0	7.5	10.0	12.5
YKL-40 ≤70.7	61 (0)	59 (0)	51 (0)	44 (1)	42 (39)	3 (42)
YKL-40 >70.7	53 (0)	50 (0)	39 (0)	32 (3)	27 (24)	4 (27)

Figure S6. Association between elevated YKL-40 and outcomes: DFS (A) and OS (B), in the subgroup of patients with normal post-adjuvant CEA, adjusted hazard ratios (HR) and 95% confidence intervals (95% CI).

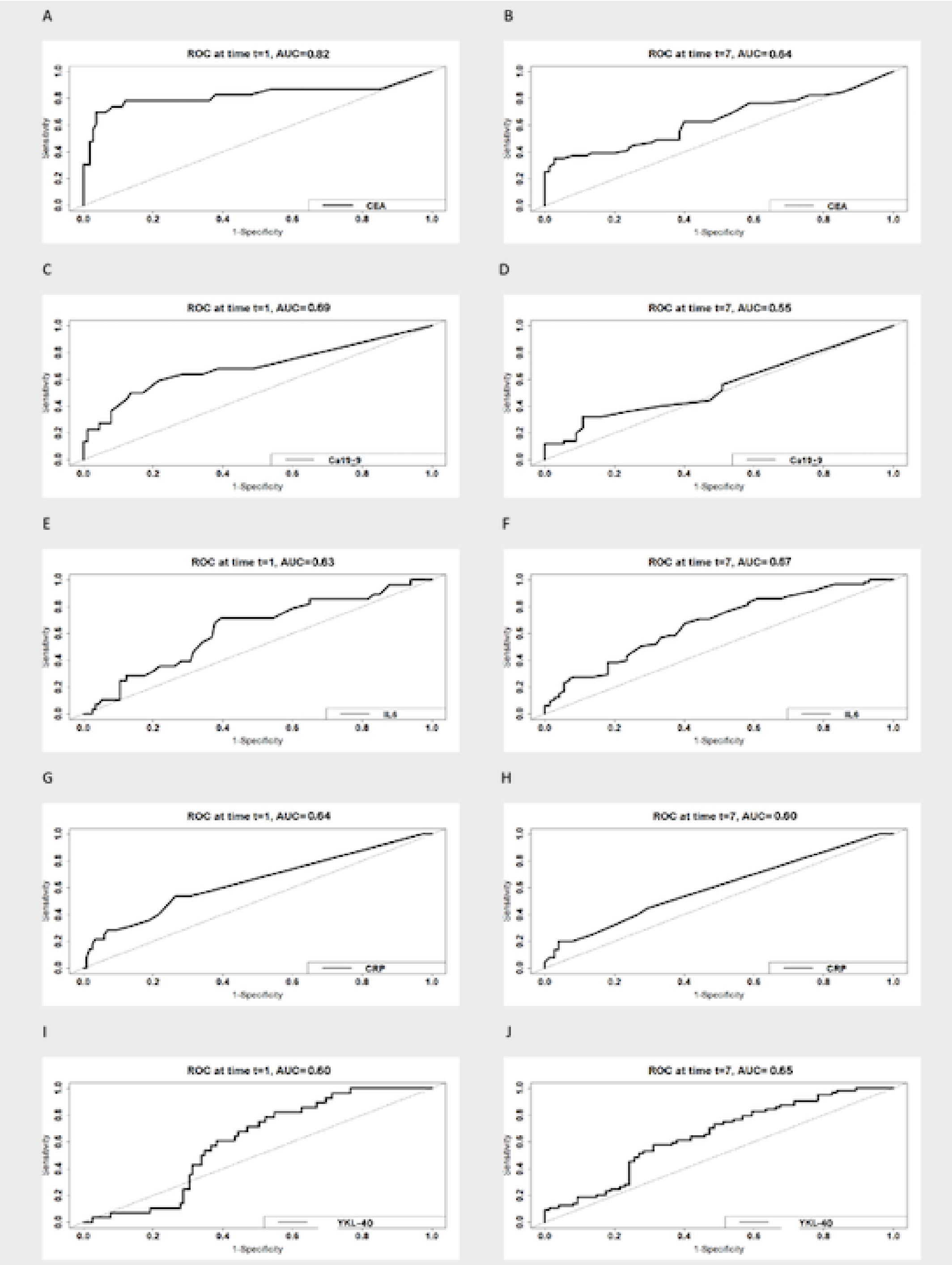

Figure S7. ROC curves depicting the accuracy for relapse with non-CRC deaths as competing event, of baseline biomarkers at 1 year (left panels) and 7 years (right panels) after the measurement of CEA (A, B), CA19-9 (C, D), IL-6 (E, F), CRP (G, H), or YKL-40 (I, J).
